# Anticancer Effects of Different Seaweeds on Human Colon and Breast Cancers

**DOI:** 10.3390/md12094898

**Published:** 2014-09-24

**Authors:** Ghislain Moussavou, Dong Hoon Kwak, Brice Wilfried Obiang-Obonou, Cyr Abel Ogandaga Maranguy, Sylvatrie-Danne Dinzouna-Boutamba, Dae Hoon Lee, Ordelia Gwenaelle Manvoudou Pissibanganga, Kisung Ko, Jae In Seo, Young Kug Choo

**Affiliations:** 1 Department of Biological Science, College of Natural Sciences, Wonkwang University, Iksan, Jeonbuk 570-749, Korea; E-Mails: strozzi37@gmail.com (G.M.); velvety7@gmail.com (D.H.K.); cyrabel@hotmail.com (C.A.O.M.); dleogns@korea.kr (D.H.L.); 2 Institute for Glycoscience, Wonkwang University, Iksan, Jeonbuk 570-749, Korea; 3 Department of Food Science and Nutrition, Keimyung University, Daegu 704-701, Korea; E-Mail: obiang@gw.kmu.ac.kr; 4 Department of Parasitology and Tropical Medicine, Kyungpook National University, Daegu 700-422, Korea; E-Mail: sylvatriez@yahoo.fr; 5 Department of Civil and Environmental Engineering, Kookmin University, Seoul 136-702, Korea; E-Mail: deliapish@gmail.com; 6 Department of Medicine, College of Medicine, Chung-Ang University, Seoul 156-756, Korea; E-Mail: ksko@cau.ac.kr; 7 College of Pharmacy, Yonsei University, Veritas D, Yonsei International Campus, Songdo-dong, Yeonsu-gu, Incheon 406-840, Korea; E-Mail: kakijane@yonsei.ac.kr

**Keywords:** breast cancer, colorectal cancer, seaweed, therapeutic compounds

## Abstract

Seafoods and seaweeds represent some of the most important reservoirs of new therapeutic compounds for humans. Seaweed has been shown to have several biological activities, including anticancer activity. This review focuses on colorectal and breast cancers, which are major causes of cancer-related mortality in men and women. It also describes various compounds extracted from a range of seaweeds that have been shown to eradicate or slow the progression of cancer. Fucoidan extracted from the brown algae *Fucus* spp. has shown activity against both colorectal and breast cancers. Furthermore, we review the mechanisms through which these compounds can induce apoptosis *in vitro* and *in vivo*. By considering the ability of compounds present in seaweeds to act against colorectal and breast cancers, this review highlights the potential use of seaweeds as anticancer agents.

## 1. Introduction

Cancers are a group of diseases characterized by uncontrolled cell growth and spread [1]. Colorectal cancer is the third most common cancer in the world, with nearly 1.4 million new cases diagnosed in 2012 [1]. Moreover, the incidence of this disease has increased steadily in recent years [2]. Despite advances in therapeutic interventions over the past few decades, the mortality rate of patients diagnosed with colorectal cancer remains approximately 40%, mainly due to metastasis to the liver [3].

Breast cancer is the leading cause of death among women in many countries [4]. This is the second most common cancer overall, with nearly 1.7 million new cases diagnosed worldwide in 2012 [1]. As breast cancer progresses, survival factors that inhibit apoptotic cell death are expressed by the cancer cells [5,6]. 

Due to the increasing incidence of cancer in both developing and developed countries, the use of new chemotherapeutic molecules is needed [7]. Employing natural or synthetic agents to prevent or suppress the progression of invasive cancers has recently been recognized as an approach with enormous potential [8].

Seaweeds (marine algae) are extensively used as functional foods and medicinal herbs, and have a long history of use in Asian countries [9]. Since certain seaweeds have long been used for the treatment of cancer, many crude or partially purified polysaccharides from various brown, green, and red algae have been tested for their antitumor activities [10] (Table 1). These studies have indicated that marine algae constitute a promising source of novel compounds with potential as human therapeutic agents. In particular, algae have been considered as a potential source of new bioactive compounds [7].

Several studies have reported that compounds extracted from seaweed may be effective anticancer agents. This review summarizes the various effects of seaweed-derived compounds on colorectal and breast cancers via promotion of cancer cell apoptosis.

**Table 1:** The effects of seaweeds and their compounds on colorectal, breast and other cancers.

Seaweed	Nature	Colorectal Cancer	Breast Cancer	Other Cancers	Therapeutic Element	IC_50_	Reference
*Fucus* sp.	Brown algae	+	+	-	Fucoidan	5–20 μg/mL	Kim *et al*. 2010 [2]
*Stypopodium* sp.	Brown algae	+	-	+	Meroditerpenoids	12.2 μM and 14 μM	Pereira *et al*. 2011 [7]
*Sargassum muticum*	Brown algae	-	+	-	Polyphenol	0.2 μg/mL	Namvar *et al*. 2013 [9]
*Ulva fasciata*	Green algae	-	-	+	Flavoids	200 μg/mL	Ruy *et al*. 2013 [11]
*Laminaria* sp.	Brown algae	+	-	-	Laminarin		Park *et al*. 2012; 2013 [12,13]
*Laurencia* sp.	Red algae	+	-	-	Dactylone	45.4 µmol/L	Federov *et al.* 2007 [14]
*Ishige okamurae* *Phoma herbarum*	Red algae	-	-	+	Ethanol extracts		Kim *et al*. 2009 [15]
*Lithothamnion* sp.	Red algae	+	-	-	Cellfood *		Aslam et *al*. 2009 [16]
*Porphyra dentata*	Red algae	-	+	-	Sterol fraction	48.3 µg/mL	Kazlowska *et al*. 2013 [17]
*Cymopolia barbata*	Green algae	-	+	-	CYP1 inhibitors	19.82 μM and 55.65 μM	Badal *et al*. 2013 [18]
*Lophocladia* sp.	Red algae	-	+	-	Lophocladines	3.1 μM and 64.6 µM	Gross 2006 [19]
*Ascophyllum nodosum*	Brown algae	-	+	-	Fucoidan		Pavia *et al*. 1996 [20]
*Gracilaria termistipitata*	Red algae	-	-	+	Methanol extracts		Ji *et al*. 2012 [21]
*Enteromorpha intestinalis* *Rhizoclonium riparium*	Green algae	-	-	+	Methanol extracts	309.05 µg/mL and 506.08 µg/m	Paul *et al*. 2013 [22]
*Aspergillus* sp.	Brown algae	-	-	+	Gliotoxin		Nguyen *et al*. 2014 [23]
Undaria pinnatifida	Brown algae				Fucoidan		Yang *et al*. 2013 [24]

**+**: effects reported; -: no effects reported; IC_50_ = the half-maximal inhibitory concentration; Seaweeds with two IC_50_ values had two different therapeutic compounds tested; * Cellfood designates all of the mineral salts and trace elements extracted from seaweeds.

## 2. Seaweed and Colorectal Cancer

Colorectal cancer is one of the most common cancers in men and women and it is particularly prevalent in developed countries. The worldwide incidence of this cancer has increased steadily in recent years, and this has been attributed to rapid changes in dietary patterns and preferences. Dietary habits can influence the risk for colorectal cancer [25], and the identification of food components that can prevent the tumorigenic process may contribute to the development of effective anti-colorectal cancer agents [2]. Many investigations have aimed to find effective ways to combat colorectal cancer. Some studies have reported that colorectal cancer can be successfully treated with marine natural products, which contain an abundance of biologically active substances with novel chemical structures and favorable pharmacological activities [11]. 

The inhibition of apoptosis in colorectal cancer cells enhances tumor growth, promotes neoplastic progression, and confers resistance to cytotoxic anticancer agents [26]. Thus, bioactive compounds that induce apoptosis in cancer cells can be used as agents for cancer chemoprevention and/or chemotherapy [2]. Accumulating evidence suggests that bioactive compounds extracted from algae produce anticancer effects through multiple mechanisms of action, including inhibition of cancer cell growth, invasion, and metastasis, and through the induction of apoptosis in cancer cells [27]. Apoptosis may be initiated either by an intrinsic (mitochondrial-mediated) pathway or by an extrinsic (death receptor-mediated) pathway [28,29,30]. Each of these pathways involves the activation of caspases and ultimately leads to apoptosis [12].

**Table 2:** Seaweed-derived compounds and their effects on apoptosis.

Therapeutic Compounds (Seaweed)	Action Site						References
	Cell Cycle Arrest	Mitochondrial Membrane	Caspases or Cyclins	GFR	P53	Pro- or Anti-Apoptotic Proteins	
Fucoidan (*Fucus* sp.)	+	+	+	+		+	Kim *et al*. 2010 [2]/Xue *et al*. 2012 [31]
Laminarin (*Laminaria* sp.)	+	+	+	+		+	Park *et al*. 2012; 2013 [12,13]
Dactylone (*Laurencia* sp.)	+	-	+	-	+		Fedorov *et al*. 2007 [14]
Steorol fraction (*Porphyra dentata*)	+	-	-	-	-	-	Kazlowska *et al*. 2013 [17]
Methanol extracts (*Sargassum muticum*)	+	-	-	-	-	-	Paul *et al*. 2013 [22]

+: effects reported; -: no effects reported.

Consumption of various types of seafood, including seaweed, has been suggested to be responsible for the low incidence of cancer in Japan and in other countries whose inhabitants traditionally consume high levels of marine organisms [14]. Numerous studies have examined the effects of seaweeds on apoptotic pathways (Table 2). The effects of laminarin, a storage glycan composed of β-glycan (β-1,3-β-1,6-glycan) found in brown algae, on colorectal cancer cells were investigated as well as the mechanisms through which laminarin induced apoptosis in these cells. Treatment with laminarin from *Laminaria* spp. (brown algae) inhibited the proliferation of colon cancer cells via Fas and IGF-IR signaling through the intrinsic apoptotic and ErbB pathways, respectively (Table 3) [12,13]. According to previous studies, Fas and Fas receptors induced the activation of members of the caspase family, leading to cleavage of apoptosis markers such as poly (ADP-ribose) polymerase (PARP) [32]. Other studies showed that laminarin regulated Fas and FADD protein levels, suggesting that it induced Fas-mediated apoptosis. Laminarin also increased the expression of Fas and FADD, which also increased the activation of caspases [33,34].

**Table 3:** Properties of seaweeds in fight against colorectal cancer.

Colorectal Cancer		References
Seaweeds	Therapeutic compounds and their properties	
*Laminaria digitata*	Laminarin from *Laminaria digitata* induced apoptosis in HT-29 colon cancer cells; affected insulin-like growth factor (IGF-IR); decreased mitogen-activated protein kinase (MAPK) and ERK phosphorylation; decreased IGF-IR-dependent proliferation	Park *et al*. 2012; 2013 [12,13]
*Lithothamnion calcareum* (Pallas), also known as *Phymatolithon calcareum* (Pallas)	Multi-mineral extract from *Lithothamnion calcareum* can protect mice on a high-fat diet against adenomatous polyp formation in the colon	Aslam *et al*. 2009 [16]
*Cymopolia barbata*	Prenylated bromohydroquinones (PBQs) isolated from *Cymopolia barbata* show selectivity and potency against HT-29 cells and inhibit CYP1 enzyme activity, which may be a lead in chemoprevention	Badal *et al*. 2012 [18]
*Undaria pinnatifida*	Fucoxanthin from *Undaria pinnatifida* attenuated rifampin-induced CYP3A4, MDR1 mRNA and CYP3A4 protein expression	Yang *et al*. 2013 [24]

ErbB receptors control key pathways that govern cellular processes such as proliferation, cell migration, metabolism, and survival [35,36]. Dysregulation of the ErbB receptor signal transduction pathway is observed in several types of cancer, including colon cancer. Abnormal activation of the ErbB receptor is thought to be one of the potential causes of cancer [37]. Laminarin decreased Bcl-2 family protein expression and inhibited cell cycle progression by regulating the ErbB signaling pathway [13]. These studies also showed that treatment of HT-29 cells with laminarin inhibited phosphorylation and ErbB2 expression, as well as the phosphorylation of Akt.

Fucoidan, a sulfated polysaccharide often found in brown algae, has shown a number of biological effects including anticancer activities [2]. A range of fucoidan structures and compositions exist in diverse brown seaweed species; however, in general the compound consists primarily of l-fucose and sulfate, along with small quantities of d-galactose, d-mannose, d-xylose, and uronic acid [38,39,40]. Recently, the diverse biological activities of fucoidan have been studied intensively, including its anticancer activities [31]. Many studies assessed whether fucoidan could inhibit the growth of colon cancer cells and studied the molecular pathways involved. Several studies showed that fucoidan exerted anticancer effects, including the suppression of growth [41,42,43,44,45,46]; it also decreased metastasis [43,47,48] and inhibited angiogenesis [48] in a variety of cancer cells. Fucoidan has been reported to inhibit the growth of a wide range of tumor cells [43,46]. It has also been shown to induce apoptosis in colon cancer HT-29 and HCT116 cells in a dose-dependent manner [15,49]. Kim *et al*. revealed that low concentrations of fucoidan (5–20 μg/mL) induced apoptosis of HT-29 and HCT-116 cells in a dose- and time-dependent manner. However, fucoidan showed a smaller effect on HTC116 cells than on HT-29 cells. According to Hyun *et al*. [50], fucoidan was able to induce apoptosis in HCT-15 human colon cancer cells at a concentration of 100 μg/mL. These results showed that the efficacy of fucoidan varied with the type of colon cancer cell studied. It was reported that fucoidan activated caspases, resulting in the induction of apoptosis through both death receptor-mediated and mitochondria-mediated apoptotic pathways [2].

Dactylone is representative of a new group of natural cancer-preventive agents [14]. Its chemical structure is closely related to that of sesquiterpenoids extracted from red algae *Laurencia* spp. The effects of dactylone have been studied in many cancer cell lines, including human colon cancer HCT116 cells, and the molecular mechanism underlying these effects was assessed [14]. Dactylone was able to suppress the phenotype expression of various human cancer cell lines and was shown to induce G1-S cell cycle arrest and apoptosis in tumor cells; it decreased Rb protein phosphorylation at Ser^795^, Ser^780^, and Ser^807/811^ sites, and also inhibited the expression of cyclin D3 and cyclin-dependent kinase (Cdk)4 [14]. Other studies revealed that inositol hexaphosphate, a dietary constituent found in rice, seems to act in a similar manner as dactylone. Indeed, inositol hexaphosphate has also been reported to decrease Cdk4 and cyclin D1 protein expression levels in addition to the inhibition of Rb phosphorylation at Ser^780^, Ser^807^, and Ser^811^, causing G_1_ arrest and apoptotic death of human cancers [14].

Meroditerpenoids such as plastoquinones, chromanols, and chromenes are a class of natural products consisting of a polyprenyl chain attached to a hydroquinone ring moiety, and are commonly present in brown algae (Phaeophyceae) [7]. Pereira *et al*. tested six meroditerpenoids (epitaondiol, epitaondiol diacetate, epitaondiol monoacetate, stypotriol triacetate, 14-ketostypodiol diacetate, and stypodiol) isolated from the brown algae *Stypopodium flabelliforme.* These meroditerpenoids inhibited cell proliferation in five cell lines: human neuroblastoma (SH-SY5Y), rat basophilic leukemia (RBL-2H3), murine macrophages (Raw267), Chinese hamster fibroblasts (V79), and human colon adenocarcinoma (Caco-2) cells. Overall, the compounds’ activities against all cell lines were efficient. Stypotriol triacetate showed the most inhibition of the colon adenocarcinoma cell line, Caco-2, followed by epitaondiol monoacetate and epitaondiol.

Over their lifetime, marine algae accumulate high levels of minerals from seawater [16]. The proliferation and differentiation of human colon carcinoma cell lines were assessed in the presence of a mineral-rich extract from the red marine alga, *Lithothamnion calcareum* [16]. This algal extract was as effective as inorganic calcium in both inhibition of colon carcinoma cell growth and induction of its differentiation. Both epidemiological studies [51,52,53,54,55] and interventional studies [56,57] in humans have demonstrated that calcium has the capacity to reduce polyp formation in the colon. Other studies have found that different minerals obtained from marine algae could also contribute to the reduction of polyp formation (Table 3). In another study, Aslam *et al.* [16] reported that a multi-mineral product obtained from marine algae was able to reduce colon polyp formation in C57BL/6 mice receiving either a high-fat diet or a low-fat diet [16]. Based on these results, they suggested that the effects of calcium alone could not explain the protective effects of the multi-mineral supplement and that a multi-mineral approach to colon polyp chemoprevention may prove to be more efficacious than an approach based on the use of calcium alone.

*Ulva fasciata* extract (UFE) from *Ulva fasciata* Delile (sea lettuce), which grows abundantly along costal seashores, was used to assess the mechanisms underlying the cytotoxicity of green algae [11]. The anti-proliferative effects of UFE against colon cancer cells involved induction of apoptosis. Reactive oxygen species (ROS) have been reported to regulate apoptotic signal transduction and induce depolarization of the mitochondrial membrane, leading to increases levels of pro-apoptotic molecules in the cytosol [58,59]. Rye *et al*. [11] demonstrated that UFE significantly increased ROS generation in HCT116 cells and that antioxidant-mediated scavenging of UFE-induced ROS reduced the UFE-mediated cell death. UFE was able to inhibit the growth of HCT116 human colon cancer cells by 50% at a concentration of 200 μg/mL. It induced apoptosis through alteration in Bcl-2 family protein expression, increasing mitochondrial membrane permeability, and activation of caspase 9 and caspase 3 [11].

## 3. Seaweed and Breast Cancer

Breast cancer is the leading cause of death among women in many countries [4]. Although male breast cancer is less common, a few studies have revealed that the incidence has increased over the past 25 years [60]. Scientists have aimed to treat breast cancer without harming the patient by exploiting the differences between cancerous and normal cells. Nutritional strategies have been applied to study populations with a low incidence of breast cancer. In Asia, seaweeds have been eaten for at least 5000 years [61]. These populations have a low incidence of breast cancer. Certain brown and red algae are known for their anticancer properties [10,62]. In cells treated with seaweed, apoptosis was observed [63], and the authors speculated that seaweed could be a breast cancer-preventing food.

Some studies recently evaluated the effect of a brown seaweed (*Sargassum muticum*) methanol extract (SMME) on the proliferation of MCF-7 and MDA-MB-231 breast cancer cell lines [9] by conducting morphological assessments of apoptosis, caspase assays, and chick chorioallantoic membrane (CAM) assays. These methods were used to determine the morphological alterations induced by SMME to evaluate its time-dependent effects on caspases 8, 9, and 3 by using caspase assays, and also to evaluate the anti-angiogenic effects of SMME using the CAM assay. They observed that treatment with SMME significantly decreased angiogenesis, leading them to speculate that SMME could decrease cancer cell proliferation and increase apoptosis of human breast cancer cells in a time- and dose-dependent manner (Table 4).

Fucoidan was reported to enhance the activity of natural killer (NK) cells, which have anticancer activity [64]. The effects of crude fucoidan extracted from *Fucus vesiculosus* on the growth of breast cancer have been determined *in vitro* and *in vivo* [31]. Crude fucoidan significantly reduced the number of viable 4T1 cells (a mouse tumor cell line used as a model of highly metastatic breast cancer), enhanced apoptosis, and down-regulated the expression of vascular endothelial growth factor (VEGF). The mechanisms thought to be responsible for these fucoidan-mediated effects are inhibition of the expression of Bcl-2 (Bcl-2 preserves the mitochondria integrity), survivin, extracellular signal-regulated kinases (ERKs), and VEGF, and an increase in caspase-3 activation.

**Table 4:** Properties of seaweeds in fight against breast cancer.

Seaweed	Therapeutic Compounds and Their Properties	References
*Sargassum muticum*	*Sargassum muticum* methanol extract (SMME) Induced apoptosis of MCF-7 cells; showed anti-angiogenic activity in the chorioallantoic membrane (CAM) assay; antioxidant effects	Namvar *et al*. 2013 [9]
*Fucus vesiculosus*	Fucoidan (sulfated polysaccharide derived from brown algae) Decreased the viable number of 4T1 cells; induced apoptosis; down-regulated VEGF expression In colon cancer reduced in viable cell numbers and induced apoptosis of human lung carcinoma A549 cells as well as colon cancer HT-29 and HCT116 cells	Xue *et al*. 2012 [31]/ Kim *et al*. 2010 [2]
*Porphyra dentata*	Sterol fraction (containing cholesterol, β-sitosterol, and campesterol) from *Porphyra dentata* Significantly inhibited cell growth *in vitro* and induced apoptosis in 4T1 cancer cells; decreased the reactive oxygen species (ROS) and arginase activity of MDSCs in tumor-bearing mice	Kaslowska *et al*. 2013 [17]
*Lophocladia* sp.	Lophocladines A and B are 2,7-naphthyridine alkaloids from *Lophocladia sp.* Lophocladine A has affinity for NMDA receptors and is a *δ*-opioid receptor antagonist; Lophocladine B was cytotoxic to NCI-H460 human lung tumor cells and MDAMB-435 breast cancer cells	Gross *et al*. 2006 [19]

A sterol fraction extract of *Porphyra dentata*, an edible red alga used as a folk medicine in Asia, was evaluated for its effects on myeloid derived suppressor cells (MDSCs) in 4T1 cancer cells (Table 4) [17]. Previous findings indicated that phytosterols such as β-sitosterol, either alone or in combination with campesterol, may offer protection from various tumors [65,66,67]. MDSCs play an important role in tumorigenesis [18,19,20,68,69]. The authors associated the anticancer activity of *Porphyra dentata* with the presence of β-sitosterol and campesterol, which reduced the suppressive activity of MDSCs and consequently decreased tumor size. These two mechanisms might affect phytosterol-related downregulation of the suppressive activity of MDSCs, which is related to their ROS accumulation and arginase activity.

## 4. Conclusions

Many studies have explored the use of seaweed in the fight against several diseases, including colorectal and breast cancers. Various therapeutic compounds from seaweed are able to induce apoptosis through different pathways and molecular mechanisms. Several studies indicated that fucoidan was able to induce apoptosis, inhibit angiogenesis, and suppress lung metastasis of breast cancer *in vitro* and *in vivo* [21,22,23,24,31,70]. Furthermore, fucoidan inhibited growth and induced apoptosis of HT-29 colon cancer cells [2]. Laminarin induced apoptosis through the Fas and IGF-IR signaling pathways and through the intrinsic apoptotic and ErbB pathways [29]. This review highlights the importance of seaweed in the fight against colorectal and breast cancer.
